# Estimation of Alpine Skier Posture Using Machine Learning Techniques

**DOI:** 10.3390/s141018898

**Published:** 2014-10-13

**Authors:** Bojan Nemec, Tadej Petrič, Jan Babič, Matej Supej

**Affiliations:** 1 Department of Automation, Biocybernetics and Robotics, Jožef Stefan Institute, Ljubljana 1000, Slovenia; E-Mails: tadej.petric@ijs.si (T.P.); jan.babic@ijs.si (J.B.); 2 Faculty of Sport, University of Ljubljana, Ljubljana 1000, Slovenia; E-Mail: matej.supej@fsp.uni-lj.si; 3 Faculty of Mathematics, Natural Sciences and Information Technologies, University of Primorska, Koper SI-6101, Slovenia

**Keywords:** alpine skiing, GNSS measurements, Inertial Measurement Unit (IMU) measurements, statistical models, LWPR, neural networks

## Abstract

High precision Global Navigation Satellite System (GNSS) measurements are becoming more and more popular in alpine skiing due to the relatively undemanding setup and excellent performance. However, GNSS provides only single-point measurements that are defined with the antenna placed typically behind the skier's neck. A key issue is how to estimate other more relevant parameters of the skier's body, like the center of mass (COM) and ski trajectories. Previously, these parameters were estimated by modeling the skier's body with an inverted-pendulum model that oversimplified the skier's body. In this study, we propose two machine learning methods that overcome this shortcoming and estimate COM and skis trajectories based on a more faithful approximation of the skier's body with nine degrees-of-freedom. The first method utilizes a well-established approach of artificial neural networks, while the second method is based on a state-of-the-art statistical generalization method. Both methods were evaluated using the reference measurements obtained on a typical giant slalom course and compared with the inverted-pendulum method. Our results outperform the results of commonly used inverted-pendulum methods and demonstrate the applicability of machine learning techniques in biomechanical measurements of alpine skiing.

## 1. Introduction

High precision Global Navigation Satellite System technology (GNSS) has been effectively used to support many outdoor measurements in sports [1–3], including alpine skiing [4–6]. One of the main reasons is the practically unlimited measurement area and real-time availability of the results. By contrast, optical measurement systems consisting of several calibrated cameras require precise calibration, post-processing and can capture motion only in a limited area. This is problematic, especially in sports like alpine skiing. On the other hand, GNSS measurements are usually restricted to capturing a motion of a single point, defined with the location of the GNSS antenna on the skier's body. Moreover, the antenna of the GNSS receiver cannot be attached to an arbitrary joint, due to satellite visibility. In order to overcome this problem, GNSS measurement setup has often been combined with Inertial Measurement Unit (IMU) based system in order to retrieve full body motion [4,7]. Although this could be a satisfactory solution, it has several limitations. The IMU system requires precise calibration and synchronization with the GNSS measurement system. Probably the most demanding aspect is the preparation and placement of IMU sensors on the subject's body, which is tedious and time-consuming to perform on the ski slope. This is a severe limiting factor in biomechanical measurements of alpine skiing, especially when many subjects are involved. Therefore, a question arises as to whether we could simplify on-site measurements by avoiding the use of IMU sensors and still obtain valuable biomechanical results of alpine skiing.

Many important parameters (qualifiers) of alpine-skiing can be estimated by tracking only a few points of the skier's body. In many cases, it is sufficient to know the location of the skier's center of mass (COM) [8–11]. Unfortunately, we cannot directly measure COM using GNSS, because this is not a fixed point, and it might not lie within the skier's body. Usually, the antenna of the GNSS system is placed behind the skier's neck or on the top of the skier's helmet [4,6]. One solution to the above problem was proposed by [12], where the skier's body was approximated with a statically balanced inverted pendulum. Kalman filtering was used to estimate accelerations necessary to compute the inverted pendulum equilibrium pose. This approach was enhanced by also taking into account the inverted pendulum dynamics and used for COM estimation in a study of air drag influence on giant slalom [13]. A similar approach based on a statically balanced inverted pendulum was proposed also in [6]. However, approximation of the skier's pose using a rigid inverted pendulum with two degrees-of-freedom is very rough and neglects important parameters of the skiing, such as the frontal and lateral flexion of the skier's body.

The aim of this study is to go beyond the current state-of-the-art and establish a novel methodological approach based on machine learning techniques that approximate the skier's body with nine degrees-of-freedom. We implemented two machine learning techniques, one well-established method based on back propagation neural networks (NN) and one modern statistical generalization method based on locally weighted projection regression (LWPR). We applied both methods on 18 runs of a typical giant slalom course and compared the results with the inverted pendulum approach proposed by [13] and with the reference data that were obtained with a combination of GNSS and IMU measurement systems [4].

## 2. Methods

### 2.1. Subjects

Five highly skilled skiers, two females and three males, participated in this study Their average age was 22.4 years (SD = 3.4 years), height 171 cm (SD = 4.3 cm), body mass 65.3 kg (SD = 6.3 kg) and total body mass with equipment 80.0 kg (SD = 6.8 kg). Prior to their participation, the subjects were informed about the course of the study and were required to sign an informed consent approved by the ethics committee of the Faculty of Sport in Ljubljana.

### 2.2. Measurement Protocol

We measured the global position and skier pose on a typical giant slalom course consisting of 12 gates. The length of the course was 350 m with an altitude difference of 99 m. Altogether, 18 ski runs were captured (3 to 4 runs for each skier).

In order to obtain training data for both the NN- and LWPR-based methods and for evaluation of the results obtained with all of the proposed methods, reference measurements that are comprised of both GNSS antenna trajectories and skier's pose were required. For a reference system, we used a differential GNSS measurement setup (Leica 1200 Series, Leica Geosystems AG, Heerbrugg, Switzerland). The measurement errors, as specified by the manufacturer for the real-time kinematics (RTK) mode, are below 10 mm for horizontal and 20 mm for vertical position accuracy with 99.99% reliability. Alpine skiing environment experiments demonstrated a 95% confidence interval at errors below 7 mm for horizontal coordinates and below 16 mm for vertical coordinates. The maximal norm of the position error encountered was 2.9 cm [4]. The sampling frequency was 20 Hz. More details about the GNSS system can be found in [14]. The GNSS measurement setup was combined with an IMU motion capture suit (Xsens MVN , Xsens Technologies, Enschede, The Netherlands) for capturing the full-body skier's posture as in previous studies [4,15]. The IMU motion capture suit (MVN) had 17 motion-tracking sensors. Each sensor measures 3D acceleration, 3D rate of turn and 3D Earth-magnetic field data. MVN was capable of tracking 22 body segments with a sampling frequency of 120 Hz. The 3D orientation accuracy of the MVN system is below 0.5 degree; measurement resolution is 0.05 degree; accelerator resolution is 0.002 g; and gyroscope resolution is 0.6 degree/s. The isolated inertial sensor's drift in the 10-s forced pendulum showed only 0.8 degree, while in the 35-s test, the drift was higher, between 2.1 degree and 4.2 degree for frequencies of 0.5–2 Hz; the position drift of the complete MVN suit without using and external GNSS was shown to be 0.2% [4]. In another study, the MVN system was compared to the optical measurement system [15]. It was shown that the mean difference between the optical system and the MVN inertial measurement system was between 0.7 degree end 4.9 degree for different analyzed body angles.

**Figure 1.**
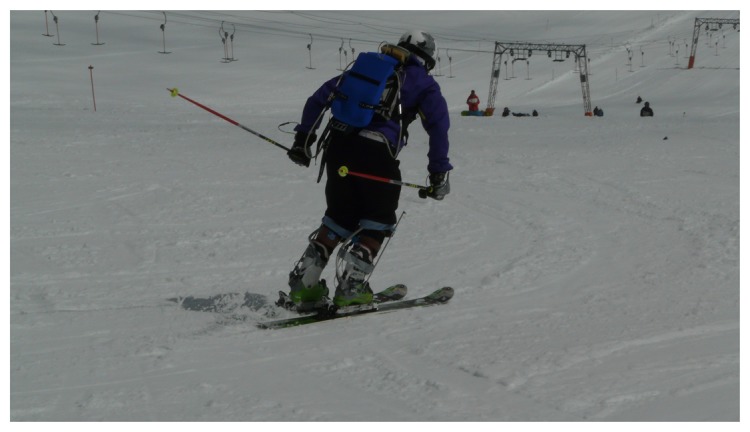
A skier equipped with the entire measurement setup consisting of GNSS and IMU units.

To combine the data from both systems, a cubic-spline interpolation was used to upscale the GNSS trajectory data from 20 to 120 Hz. The MVN data were expressed relative to the GNSS antenna coordinates; therefore, simple addition of the data from both systems gives the global position of each body segment. The data of both systems were time synchronized by an isolated explosive squat movement prior to each run. Therefore, the data recorded from the motion capture suit and from the GNSS were imported to the data analysis system, where the isolated explosive squat movement was found automatically from the motion capture suit and the GNSS. The lowest position of the squat was used as the synchronization point. An optimization process was used to calculate where the vertical velocity of the GNSS and the MVN suit's neck joint crossed the zero point [4]. The overall setup forms a reliable system for 3D measurements that is highly applicable in alpine skiing [4] due to the practically unlimited measurement volume. A skier wearing the entire setup for measuring skiing data is presented in Figure 1.

In this study, the errors were calculated as the Euclidean norm of the distances between the measured values from the reference setup and the estimated values from the model quoted for all 3 dimensions in the Cartesian space. For this, all ski runs were normalized to equal duration and expressed as a function of the ski path *s* = {0, 1}, 0 denoting the start and 1 the end of the ski run, respectively. This technique assured that the errors from the individual ski runs were summed along the ski path in order to calculate mean values and the corresponding standard deviations.

### 2.3. Estimation by the Inverted Pendulum Model

**Figure 2.**
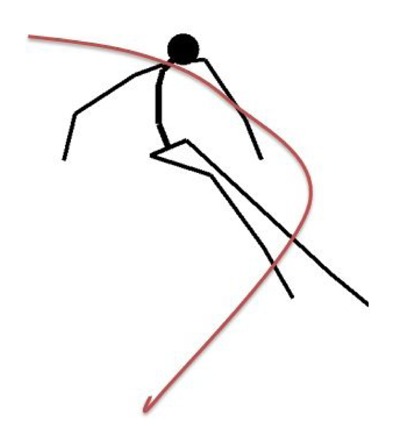
Simplified skeleton model of a skier. The red line denotes the antenna path measured with a GNSS setup.

**Figure 3.**
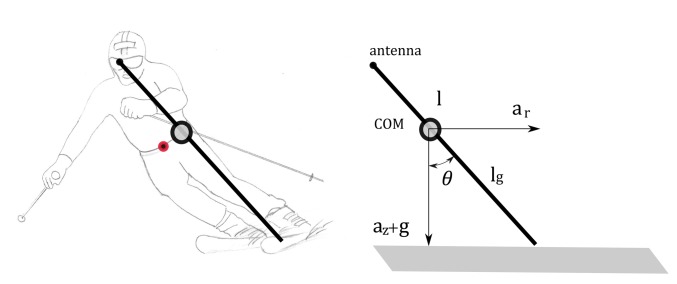
Skier modeled as the inverted pendulum. The red circle on the skier and the black circle on the model denote the center of mass (COM) of the skier and COM of the model, respectively.

The aim of the inverted pendulum model (IP) is to estimate the center of mass (COM) and the skis' trajectories from the GNSS trajectory of the skier. In our setup, the antenna that captures the position is located behind the skier's neck at shoulder height. Retrieval of even a simplified multi-segment body model, as shown in the Figure 2, from a single point is an underdetermined problem, with an infinite number of possible solutions for a given measured (captured) GNSS point. The problem becomes tractable by simplifying the body model as a single bar mechanism and by implementing a constraint that this bar is constantly dynamically balanced, as shown in Figure 3. This problem corresponds to finding the bottom motion trajectory of an unactuated inverted pendulum on a cart with a given motion trajectory of the pendulum top. The dynamics of an inverted pendulum is given by:

(1) $$J\ddot{\theta }+l_{g}\times F_{g}-l_{g}\times F_{r}=\tau ,$$

where *J* denotes the moment of inertia of the inverted pendulum, θ is the angle between the pendulum and the global Z coordinate, *l_g_* is the center of the mass of the pendulum and *F_r_* and *F_g_* are the radial and gravitational forces, respectively. τ is torque acting on the inverted pendulum and is supposed to be much lower compared to the radial and gravitational forces. Therefore, τ was set to zero in further analyses. The trajectory of the center of mass in the *X* – *Y* plane can be at any point parameterized with the curvature radius *r* and the angular velocity ω around the global *Z*-axis. Thus, the radial force is *F_r_* = *ma_r_*, and the gravity force is *F_g_* = *mg*, where *g* denotes gravity acceleration and *a_r_* is the radial acceleration, *a_r_* = ω*r*. Inserting radial and gravitational force into Equation (1) and assuming point mass at the COM for the pendulum inertia yields:

(2) $$l_{g}\ddot{\theta }+a_{r}\text{cos}(\theta )-(a_{z}+g)\text{sin}(\theta )=0.$$

Our pendulum model has thus 2 degrees of freedom, angle θ and length to the center of mass *l_g_*. Length to the center of the mass is related to the total length *l* by the *l_g_* = *kl*, where *k* is a constant that denotes the average ratio of the center of the mass to the standing height for humans. This factor varies from 0.57 to 0.55 for females and males, respectively [16]. Equation (2) can be solved for θ using tangent half-angle substitution [17] and assuming that the acceleration θ̈ is a known parameter.

(3) $$\theta =2\;\text{arctan}(a_{z}+g+\frac{\sqrt{a_{r}^{2}+{\left(a_{z}+g\right)}^{2}-l_{g}^{2}{\ddot{\theta }}^{2}}}{l_{g}\ddot{\theta }-a_{r}}).$$

**Figure 4.**
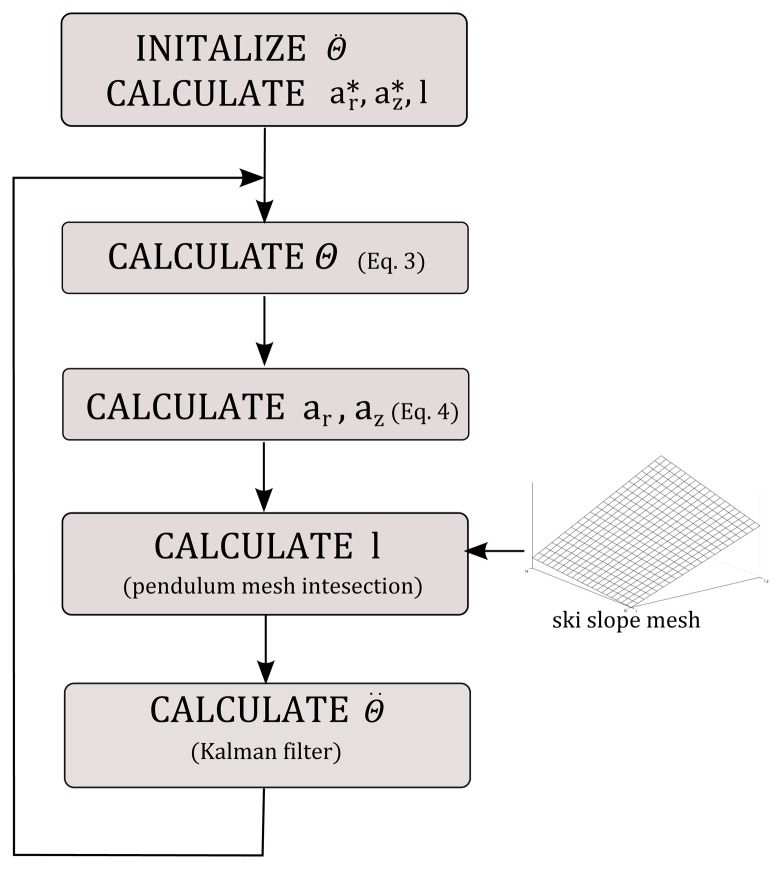
Block diagram of optimization procedure for estimating skier's posture using the inverted pendulum model.

However, Equation (3) cannot be solved directly, because neither θ̈ nor *l_g_* are known in advance. However, if the trajectory of the antenna is known, it is possible to estimate radial accelerations $a_{r}^{*}$ and vertical accelerations $a_{z}^{*}$, which corresponds to the position of the antenna. Note that the position of the antenna also corresponds to the top of the pendulum in our model. The relation between the COM accelerations and antenna accelerations is given with:

(4) $$\begin{array}{ll} a_{r} & =a_{r}^{*}+k\sin(\theta )(\dot{l}-l{\ddot{\theta }}^{2}+\omega )+k\cos(\theta )(l\dot{\theta }+2\dot{l}\dot{\theta })\\ a_{z} & =a_{z}^{*}+k\cos(\theta )(\dot{l}-l{\ddot{\theta }}^{2})-k\sin(\theta )(l\dot{\theta }+2\dot{l}\dot{\theta }) \end{array}$$

which, again, requires known pendulum angle θ, pendulum length *l* and their derivatives. Therefore, the optimal solution can be found by applying an optimization method. In the optimization procedure, the initial angular accelerations θ̈ are set to zero in order to calculate antenna accelerations $a_{r}^{*}$, $a_{z}^{*}$ and angular velocities ω for the entire trajectory. Calculation of angular velocities is accomplished through the estimation of the curvature radii of the circles spanned through three adjacent trajectory points. From the first approximation of COM accelerations with the accelerations of the antenna, the pendulum angle θ and the pendulum length are calculated along the entire trajectory. Pendulum length is calculated from the intersection of a vector, defined with the antenna position and angle θ, and measured ski slope mesh. From measured θ, the calculation of θ̈ is performed using Kalman filtering, which optimally estimates accelerations from the noisy measurements [12]. The whole procedure repeats until it converges. Typically, it requires from 5 to 10 iterations until it converges to the final solution. The entire optimization procedure shown in the block diagram in Figure 4 was implemented in MATLAB (Mathworks Inc., Natick, MA, USA).

### 2.4. Estimation by Back Propagation Neural Networks

Estimation of the skier's pose using the inverted pendulum model is a very rough approximation. On the other hand, an average skiing coach can predict the pose of the skier based on relevant parameters, such as velocity, ski turn phase, ski slope inclination, and others. The hypothesis is that it is possible to build a model of the skier capable of predicting the skier's pose based on some easy measurable parameters, for example the trajectory of the GNSS antenna and local ski slope inclination, which can be both directly measured with GNSS technology.

**Figure 5.**
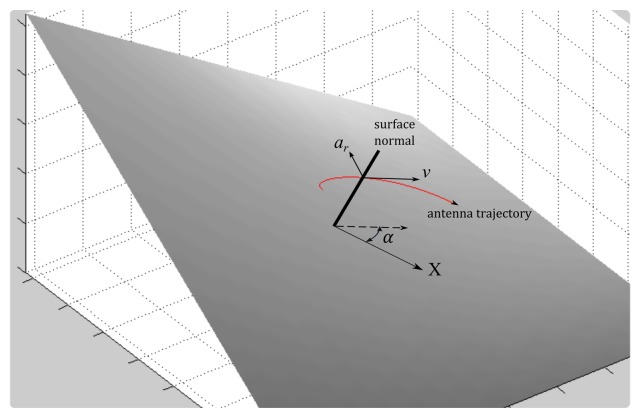
Input (query) of skiing pose prediction model: radial acceleration of the antenna *a_r_*, absolute velocity *v* and the angle α between the local skiing direction and *X*-axis of the ski slope surface normal.

**Figure 6.**
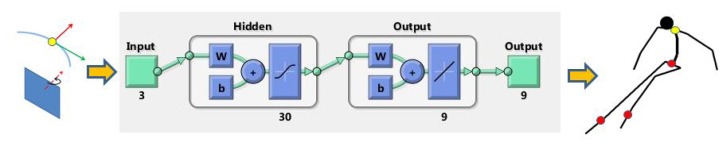
Framework for the estimation of skier's pose using artificial neural networks.

One possibility for building a model of a skier are artificial neural networks (NN), which are computational models inspired by animal and human central nervous systems [18,19]. They consist of interconnected computational models of neurons and compute output values as a function of inputs and interconnection weights. Neural networks have been successfully applied to solve a wide variety of tasks that are hard to accomplish using ordinary rule-based programming, including computer vision, speech recognition, motor control, *etc* [20]. During the learning, the set of adaptive weights were optimized in a way that a set of input queries generate the set of data describing the skier's pose. Once the NN model is adequately learned (trained), it returns the most probable configuration of the skier's pose regarding the input data, referred to as the query. The query was chosen to be a three-dimensional vector consisting of the radial acceleration of the antenna *a_r_*, absolute velocity *v* and the angle α, which was calculated as the angle between the local skiing direction and the *X*-axis of the ski slope surface normal (see Figure 5). The output set of data was a 9-dimensional vector consisting of the COM position, the left ski position and the right ski position, all expressed relative to the GNSS antenna position, respectively. Each position data was a 3D vector of *x,y* and *z* coordinates. The entire procedure is schematically outlined in Figure 6.

Neural networks were implemented in MATLAB using the Neural Networks Toolbox. The main tuning parameter in this setup was the number of hidden layers [18]. Too many hidden layers can cause the overfitting problem, slow down the learning and could even degrade the performance of the NN [21]. Similarly, too few hidden layers cannot cope with the nonlinearity of the approximated function. In our case, the best results were obtained with 30 hidden layers resulting in a NN with 399 neurons.

### 2.5. Estimation by Statistical Generalization

**Figure 7.**
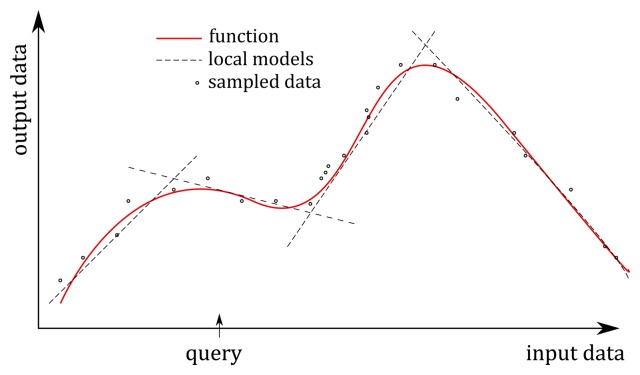
An example of function approximated with local linear models.

In NN, as well as in most learning methods, a single global model is used to fit all of the training data. Recently, new approaches were proposed based on multiple local models that attempt to fit the training data only in a region around the location of the query [22,23]. Among the benefits of the these methods are inexpensive computation, on-line learning capability and generally more accurate modeling of highly non-linear systems [24]. As such, they are suitable for building a skier model. In our research, we applied a statistical generalization method called locally weighted projected regression (LWPR) [24,25], where the aim was to find a set of output data that correspond to the input data, which was given as a query [26]. It is a non-parametric regression method that combines multiple local methods for averaging, interpolating between and extrapolating from, the data associated with a particular query. The key concept of the method was to approximate nonlinear functions by means of piecewise linear models (see Figure 7), where the main problem was to determine the region of validity of the local models and how to fit the local model in this region. The detailed description of the applied algorithm is beyond the scope of this paper and can be found in [25]. In our experiments, we used the Open-Code MATLAB implementation of the LWPR algorithm [27]. The main tuning parameter in this implementation turned out to be the positive semi-definite distance metric matrix **D** that determined the size and shape of region of validity of the linear models and influenced the number of applied local models [25]. Higher diagonal values of this distance metrics matrix increased the number of local models. Too many local models caused overfitting, which can drastically degrade the performance of the LWPR model in our case. The best results were obtained with **D** = 0.1**I**, where I denotes the 3 × 3 identity matrix. This choice resulted in 50 linear models, which were enough to predict the skier's pose given the input set of the data. As in the case of NN, the input query was chosen to be a three-dimensional vector consisting of the radial acceleration of the antenna *a_r_*, the absolute velocity *v* and the angle α, and the output set of data was a 9-dimensional vector consisting of the COM position, the left ski position and the right ski position, all expressed relative to the GNSS position of the antenna, respectively.

## 3. Results

**Figure 8.**
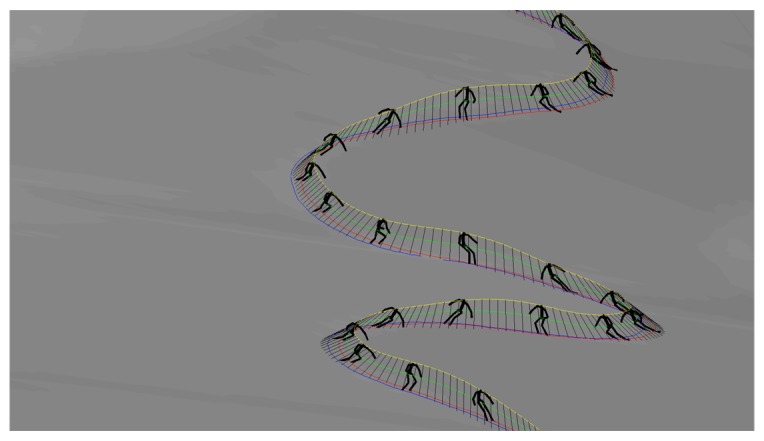
A comparison of inverted pendulum model (black line) and measured pose based on a 22 segment model. Yellow, green, red and blue color denote the antenna, COM, right ski and left ski trajectories, respectively.

**Figure 9.**
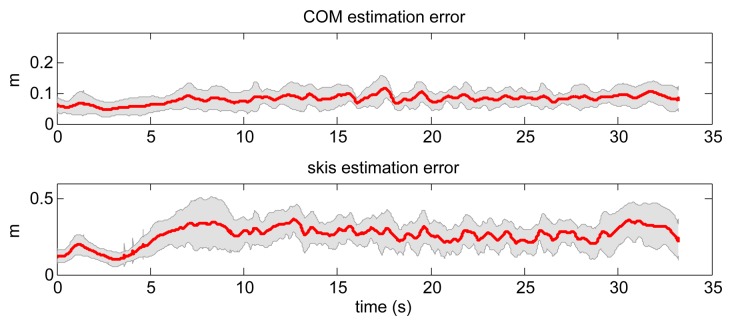
Mean error between estimated and true values of COM position and the position of skis using the inverted pendulum model for all 18 ski runs. The shadowed region denotes the standard deviation of the mean error.

First, we evaluated the inverted pendulum method by estimating the position of the COM and the bottom of the pendulum for all 18 ski runs using only GNSS data. The results were compared to the reference data obtained with the GNSS-IMU setup. A direct comparison was possible only for the COM estimation, since the estimated position of the bottom of the pendulum cannot be directly compared to the positions of the skis. Therefore, we compared the mid-point between the skis and the bottom of the pendulum by assuming that the weight is equally distributed between both skis. A snapshot of a typical ski turn (ski run Number 4) is shown in Figure 8, where we can see both measured skier's poses and the inverted pendulum approximations indicated with the black bar. Mean values and the corresponding standard deviations of the estimated position errors of the COM and the positions of the mid-skis for all 18 runs are shown in Figure 9. As expected, estimation of the skis position is less accurate then estimation of COM. It can be noticed also that the inverted pendulum model exhibits larger error in the initial phase of the turn, which is evident also from Figure 8.

**Figure 10.**
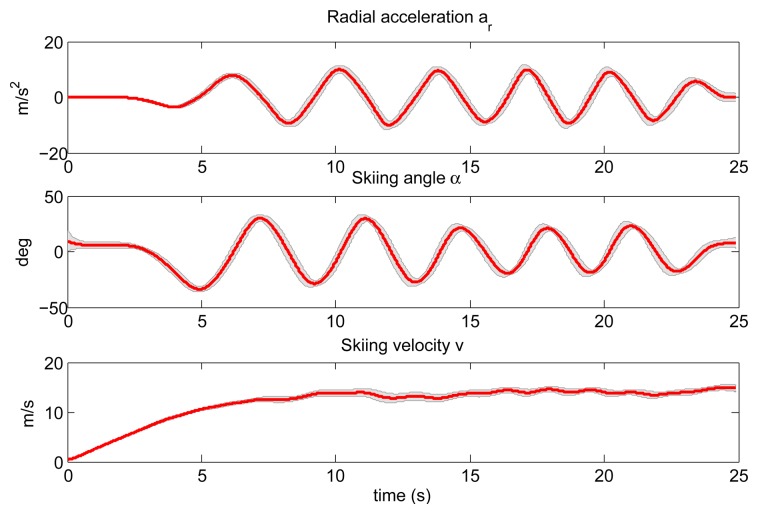
Mean values of query data composed of radial acceleration *a_r_*, skiing angle α and skiing velocity *v* for all 18 ski runs. The shaded region denotes the standard deviation.

Next, we evaluated the NN model. The NN model was trained using three ski runs, one performed by the female subject and two performed by male subjects. Using the trained neural network and GNSS data as the query, we estimated the position of the COM and the position of the both skis for all 18 ski runs. Query data composed of radial acceleration *a_r_*, skiing angle α and skiing velocity *v* are shown in Figure 10. To obtain these plots, we normalized all ski runs from the start point to the finish point to equal duration and calculated mean values and the corresponding standard deviations for all 18 runs.

**Figure 11.**
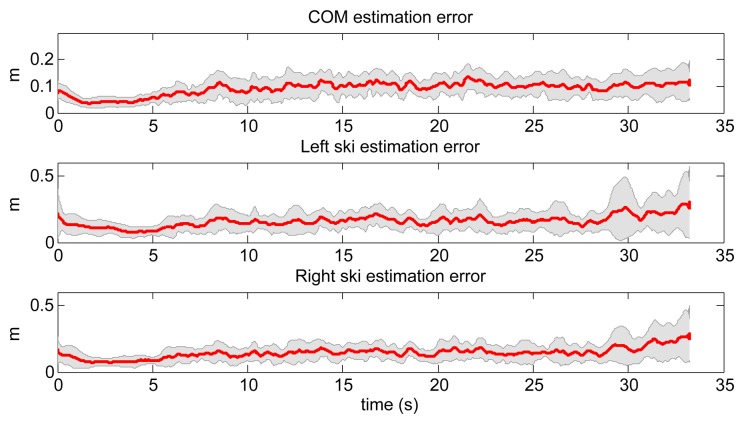
Mean error between the estimated and true value of COM positions and both skis positions by using NN for all 18 ski runs. Yellow, green blue and red lines denote the antenna trajectory, COM trajectory and left and right skis trajectories, respectively. Measured trajectories are solid lines, and estimated ones are denoted with dotted lines.

The results were compared to the data obtained with the reference system, as described in Section 2.2. Mean errors of estimation of COM and both skis for all ski runs using the NN prediction model are shown in Figure 11. It can be clearly seen that the errors obtained with the NN model are significantly lower compared to the errors using the IP model.

**Figure 12.**
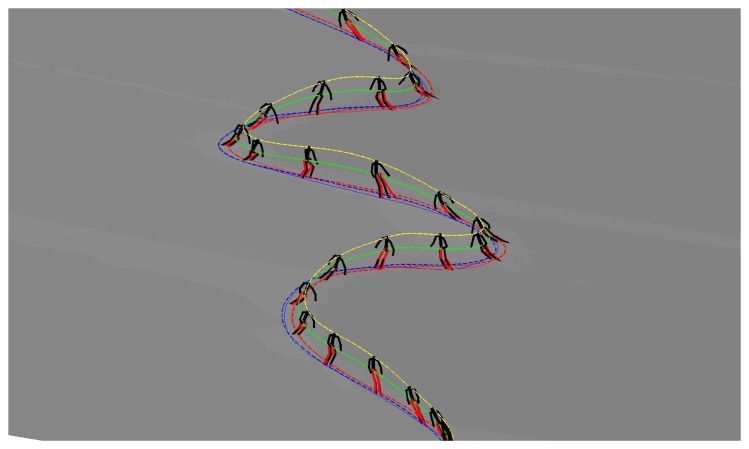
A comparison between the LWPR model (red) and measured pose (black). Note that the upper body pose was not estimated with the LWPR model.

**Figure 13.**
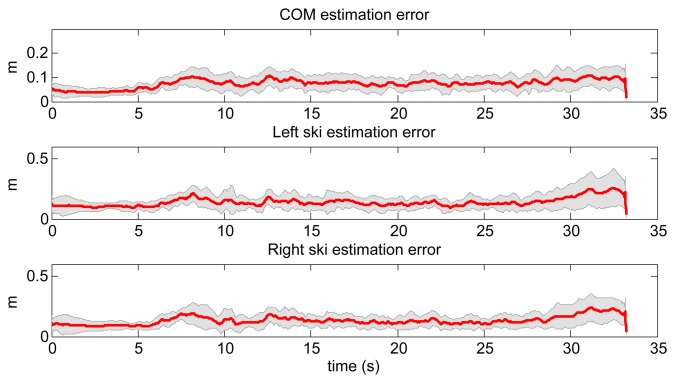
Mean errors of COM positions and skis positions, estimated by using LWPR for all 18 ski runs. The shadowed region denotes the standard deviation.

Afterwards, we trained the LWPR with the same three ski runs as for the NN based model. The same query data set as in previous case was used also for the evaluation (see Figure 10). The obtained data were compared with the measured data obtained with the reference setup. A typical ski run (Number 4) is given in Figure 12, where we can see the measured skier's pose with GNSS-IMU in black and the LWPR prediction in red. Note that the upper body of the skier, including the arms, the neck and the head, was not estimated within this study. In Figure 13, we show the estimation errors and the standard deviations of the COM position and the position of the both skies in all 18 runs. Shaded areas denote the standard deviations.

Here, the main issue arises of how general the approximation of the skier's pose was, with respect to the different subjects and different ski runs. To investigate this issue, we performed an additional cross-check validation to estimate the accuracy of the trained modes. Both the NN and LWPR models were trained individually using three runs of each subject (skier). The obtained individual models were used to predict the pose of all skiers. The prediction errors for the COM and for the ski position are shown in Tables 1, 2, 3 and 4 for NN- and LWPR-based models, respectively. In this table, columns correspond to the model, which was trained using three runs of the same skier. Rows correspond to all ski runs of each skier. As can be observed from the tables, the lowest prediction error was on the table diagonal, which denotes the case when the model was trained with the same subject that was later used for the prediction. These areas are denoted with green background color in the tables.

A paired-samples *t*-test was conducted to compare prediction errors when using NN and LWPR methods. Here, we compared only results obtained using models trained with different subjects that were later used for the prediction. On average, the COM prediction error was statistically significantly lower when using LWPR (M = 0.059 m, SD = 0.011 m) than with NN (M = 0.062 m, SD = 0.013 m), *t*(71) = 2.61, *p* = 0.01. Similarly, the prediction error of ski position was also statistically significantly lower when using LWPR (M = 0.089 m, SD = 0.018 m) than with NN (M = 0.115 m, SD = 0.023 m), *t*(71) = 10.67, *p* < 0.00.

## 4. Discussion

Until now, the inverted pendulum model was the only method used to predict the skier's pose using solely GNSS data. Although we derived a complete dynamic model of the IP, the results show that the IP model cannot accurately estimate COM and skis positions, despite the fact that other studies showed usable ground reaction forces and air drag estimation using a similar or equal methodology [6,13]. The main drawback of the inverted pendulum model is the assumption that the COM position lies on the line connecting the position of the antenna and the center of the pressure point of the ground, which is somewhere between both skies. However, the actual skier's COM almost never lies in this line during the ski turns; therefore, the differences between estimated and actual COM positions may be substantial (see Figure 3). Another issue with the inverted pendulum model is that it can only predict the one single point on the ground (center of pressure) and not the actual skis' positions. Moreover, the assumption that the skier can be approximated with a non-actuated inverted pendulum is also very rough. In reality, the skier can produce substantial torque in the frontal plane regarding the skiing direction, which violates the assumption of zero pendulum driving torque τ [28,29]. However, this torque cannot be measured with the GNSS setup.

On the contrary, modeling based on NN and LWPR account for all of these effects to some extent. Mainly, they are limited by the fact that they will always return an average, *i.e.*, the most probable pose regarding the input queries. In other words, they cannot predict the correct pose if the skier has taken an unusual pose, which was not included in the training data. Such a pose might happen, for example, when the skier tries to prevent falling after a mistake or an imbalance during skiing. On the other hand, this feature opens new possible applications of the proposed skiing models. Since they could detect the unexpected movements, e.g., mistakes, it would be possible to develop an automatic coaching process using the proposed technology. Such information would be of great interest when considering skiing safety or when analyzing competitive skiers where a small deviation from “standard” skiing could be crucial for the performance. Summarizing, the proposed skier's pose prediction based on trained NN and LWPR models is not meant to be a replacement for a complete measurement setup consisting of GNSS and IMU sensors. Rather than that, they can generate a decent approximation of the skier's pose in absence of the complete GNSS-IMU setup. Their prediction errors are substantially lower compared to those obtained with the inverted pendulum model. Additionally, they are also computationally less demanding compared to the inverted pendulum model. If real-time data streaming from the GNSS system is provided, they are able to predict the skier's pose in real time. On the contrary, the dynamic inverted pendulum method is based on the optimization and, thus, requires data of the entire ski run.

Appropriately chosen pairs of input (query) and output data are crucial for the success of the statistical generalization. It is obvious that skier's pose can not be predicted using only antenna position. Even the simplest possible model as given by the Equation (2) uses also COM accelerations. This implies that the knowledge of accelerations is also required. On the other hand, a high dimensional query limits the performance of the statistical generalization methods in general. In our case, we got the best results for the query composed of the radial acceleration of the antenna *a_r_*, absolute velocity *v* and the local skiing direction angle α, as explained in Figure 5.

NN- and LWPR-based models cannot predict completely new, previously unseen situations. For example, the models cannot correctly predict giant slalom courses at high skiing velocities if the models were trained only with special slalom courses performed at low skiing velocity. On the other hand, they are effective at predicting giant slalom and special slalom courses, if both disciplines were used in training. However, the best results are obtained if the models are trained and used for each skiing discipline individually. As we can see from Tables 1, 2, 3 and 4, the best results were obtained also in the case when the models were individually trained for the same subject. This is indeed an expected result, but the prediction errors if we use individually trained models for other subjects are very small, as well. This shows that the learned models are, to a great extent, subject independent. Additionally, we can see that slightly lower errors were obtained using the LWPR model, also confirmed with the paired-samples *t*-test. This indicates that the LWPR method is more efficient when predicting previously unseen situations.

## 5. Conclusions

By comparing both proposed learned model-based methods, we see that the LWPR-based model performed slightly better and the training time is lower compared to the NN-based model. On the other hand, both LWPR- and NN-based methods perform substantially better than the IP-based method. The main drawback of the newly proposed methods is that they require training data based on a complete setup consisting of IMU and GNSS sensors or any other full-body measurement system (e.g., a calibrated camera-based setup). Since such measurements are difficult to obtain, we propose to establish a publicly available database of measurements, where all laboratories capable of providing such measurements could contribute. The models could then be classified according to the skiing discipline and skills of the skier. The main purpose of such a database with models would go beyond research and athlete coaching; it could have a great potential to be used also in conjunction with smart phones to visualize, monitor and coach also amateur skiers.

## Figures and Tables

**Table 1.** Cross-validation of NN models. Models were learned using all runs of the same skier (columns). Mean errors and standard deviations of the COM position of these models were calculated for all 18 runs. All values in the table are given in m.

		**MODEL TRAINING / SKIER**				
SKIER	RUN	1	2	3	4	5
1	1	0.034 (0.017)	0.062 (0.025)	0.081 (0.040)	0.075 (0.061)	0.097 (0.051)
	2	0.030 (0.014)	0.049 (0.025)	0.056 (0.025)	0.040 (0.019)	0.060 (0.026)
	3	0.033 (0.018)	0.055 (0.031)	0.064 (0.027)	0.046 (0.021)	0.079 (0.038)
2	1	0.067 (0.033)	0.037 (0.017)	0.055 (0.026)	0.060 (0.033)	0.053 (0.027)
	2	0.056(0.031)	0.033 (0.017)	0.062 (0.025)	0.059 (0.025)	0.075 (0.026)
	3	0.054 (0.031)	0.029 (0.015)	0.056 (0.023)	0.054 (0.025)	0.072 (0.028)
	4	0.063 (0.035)	0.042 (0.020)	0.057 (0.021)	0.069 (0.031)	0.070 (0.027)
3	1	0.063 (0.029)	0.049 (0.024)	0.036 (0.016)	0.068 (0.030)	0.048 (0.027)
	2	0.055 (0.024)	0.049 (0.024)	0.031 (0.014)	0.059 (0.026)	0.054 (0.025)
	3	0.074 (0.027)	0.074 (0.021)	0.045 (0.018)	0.086 (0.028)	0.069 (0.024)
	4	0.064 (0.027)	0.055 (0.022)	0.037 (0.017)	0.073 (0.030)	0.047 (0.022)
4	1	0.056 (0.025)	0.046 (0.020)	0.054 (0.024)	0.037 (0.017)	0.068 (0.028)
	2	0.045 (0.019)	0.050 (0.022)	0.065 (0.021)	0.031 (0.014)	0.061 (0.024)
	3	0.048 (0.027)	0.054 (0.024)	0.070 (0.025)	0.035 (0.014)	0.064 (0.024)
5	1	0.081 (0.045)	0.067 (0.034)	0.057 (0.037)	0.095 (0.044)	0.028 (0.014)
	2	0.085 (0.055)	0.066 (0.027)	0.055 (0.028)	0.102 (0.043)	0.027 (0.014)
	3	0.065 (0.029)	0.058 (0.022)	0.046 (0.019)	0.061 (0.028)	0.031 (0.016)
	4	0.066 (0.028)	0.057 (0.021)	0.044 (0.019)	0.067 (0.029)	0.033 (0.019)
method : NN		**COM MEAN ERRORS (STANDARD DEVIATIONS)**				

**Table 2.** Cross-validation of NN models. Models were learned using all runs of the same skier (columns). Mean errors and standard deviations of the positions of the skis for these models were calculated for all 18 runs. All values in the table are given in m.

		**MODEL TRAINING / SKIER**				
SKIER	RUN	1	2	3	4	5
1	1	0.058 (0.031)	0.128 (0.056)	0.150 (0.094)	0.182 (0.205)	0.206(0.151)
	2	0.049 (0.027)	0.103 (0.046)	0.096 (0.052)	0.090 (0.036)	0.113 (0.059)
	3	0.058 (0.032)	0.128 (0.061)	0.116(0.059)	0.108 (0.044)	0.150 (0.082)
2	1	0.150(0.072)	0.061 (0.032)	0.114(0.055)	0.105 (0.058)	0.112 (0.065)
	2	0.120(0.063)	0.057 (0.030)	0.104 (0.061)	0.102 (0.046)	0.123 (0.059)
	3	0.120(0.063)	0.047 (0.023)	0.096 (0.059)	0.093 (0.044)	0.126(0.062)
	4	0.118 (0.066)	0.067 (0.036)	0.096 (0.051)	0.098 (0.050)	0.113 (0.055)
3	1	0.118 (0.058)	0.098 (0.053)	0.056 (0.026)	0.118 (0.048)	0.093 (0.053)
	2	0.101 (0.043)	0.097 (0.057)	0.043 (0.020)	0.108 (0.043)	0.094 (0.042)
	3	0.112 (0.046)	0.109 (0.046)	0.052 (0.022)	0.130 (0.036)	0.109 (0.029)
	4	0.118 (0.049)	0.106(0.053)	0.056 (0.027)	0.118 (0.049)	0.090 (0.037)
4	1	0.111 (0.041)	0.091 (0.037)	0.114 (0.042)	0.052 (0.024)	0.116(0.054)
	2	0.100 (0.040)	0.083 (0.038)	0.101 (0.036)	0.045 (0.017)	0.088 (0.042)
	3	0.110(0.045)	0.091 (0.041)	0.113 (0.046)	0.053 (0.024)	0.102 (0.049)
5	1	0.156(0.096)	0.138 (0.070)	0.105 (0.058)	0.148 (0.070)	0.052 (0.029)
	2	0.170(0.116)	0.141 (0.068)	0.101 (0.049)	0.160 (0.061)	0.043 (0.021)
	3	0.126(0.054)	0.117 (0.053)	0.095 (0.031)	0.091 (0.044)	0.051 (0.027)
	4	0.128 (0.052)	0.121 (0.058)	0.094 (0.030)	0.100 (0.051)	0.062 (0.032)
method : NN		SKI MEAN ERRORS (STANDARD DEVIATIONS)				

**Table 3.** Cross-validation of LWPR models. Models were learned using all runs of the same skier (columns). Mean errors and standard deviations of the COM positions of these models were calculated for all 18 runs. All values in the table are given in m.

		**MODEL TRAINING / SKIER**				
SKIER	RUN	1	2	3	4	5
1	1	0.042 (0.019)	0.058 (0.018)	0.076 (0.030)	0.052 (0.021)	0.090 (0.041)
	2	0.032 (0.012)	0.046 (0.022)	0.057 (0.021)	0.030 (0.012)	0.066 (0.029)
	3	0.034 (0.012)	0.047 (0.022)	0.067 (0.031)	0.041 (0.018)	0.076 (0.035)
2	1	0.074 (0.031)	0.048 (0.020)	0.073 (0.027)	0.056 (0.027)	0.065 (0.035)
	2	0.059 (0.026)	0.040 (0.014)	0.066 (0.018)	0.053 (0.020)	0.076 (0.020)
	3	0.052 (0.021)	0.031 (0.013)	0.058 (0.026)	0.041 (0.020)	0.063 (0.027)
	4	0.054 (0.020)	0.041 (0.013)	0.051 (0.013)	0.054 (0.020)	0.064 (0.019)
3	1	0.063 (0.025)	0.050 (0.026)	0.042 (0.021)	0.061 (0.023)	0.046 (0.014)
	2	0.050 (0.022)	0.049 (0.021)	0.042 (0.018)	0.053 (0.025)	0.050 (0.028)
	3	0.077 (0.015)	0.077 (0.020)	0.049 (0.016)	0.076 (0.025)	0.069 (0.016)
	4	0.056 (0.024)	0.057 (0.019)	0.040 (0.021)	0.057 (0.022)	0.051 (0.025)
4	1	0.055 (0.022)	0.048 (0.021)	0.056 (0.021)	0.054 (0.013)	0.062 (0.026)
	2	0.047 (0.017)	0.049 (0.029)	0.063 (0.019)	0.026 (0.015)	0.058 (0.021)
	3	0.061 (0.023)	0.059 (0.023)	0.076 (0.025)	0.034 (0.013)	0.069 (0.025)
5	1	0.071 (0.028)	0.055 (0.025)	0.063 (0.026)	0.062 (0.029)	0.049 (0.021)
	2	0.083 (0.032)	0.063 (0.026)	0.066 (0.033)	0.066 (0.031)	0.045 (0.022)
	3	0.063 (0.025)	0.064 (0.020)	0.049 (0.018)	0.058 (0.028)	0.043 (0.017)
	4	0.060 (0.025)	0.056(0.015)	0.047 (0.018)	0.055 (0.022)	0.039 (0.017)
method : LWPR		**COM MEAN ERRORS (STANDARD DEVIATIONS)**				

**Table 4.** Cross-validation of LWPR models. Models were learned using all runs of the same skier (columns). Mean errors and standard deviations of the positions of the skis for these models were calculated for all 18 runs. All values in the table are given in m.

		**MODEL TRAINING / SKIER**				
SKIER	RUN	1	2	3	4	5
1	1	0.066 (0.039)	0.118 (0.047)	0.126(0.063)	0.106(0.051)	0.132 (0.093)
	2	0.044 (0.020)	0.098 (0.030)	0.095 (0.039)	0.070 (0.029)	0.098 (0.068)
	3	0.061 (0.040)	0.110 (0.049)	0.125 (0.083)	0.089 (0.048)	0.132 (0.097)
2	1	0.139 (0.066)	0.082 (0.032)	0.126(0.077)	0.100 (0.052)	0.124 (0.097)
	2	0.097 (0.056)	0.057 (0.027)	0.085 (0.054)	0.078 (0.041)	0.099 (0.064)
	3	0.094 (0.045)	0.038 (0.021)	0.079 (0.068)	0.062 (0.032)	0.086 (0.075)
	4	0.081 (0.039)	0.056 (0.028)	0.063 (0.034)	0.057 (0.036)	0.081 (0.046)
3	1	0.099 (0.057)	0.089 (0.055)	0.063 (0.032)	0.088 (0.057)	0.071 (0.031)
	2	0.072 (0.030)	0.089 (0.052)	0.056 (0.036)	0.081 (0.051)	0.084 (0.066)
	3	0.082 (0.038)	0.092 (0.051)	0.041 (0.021)	0.083 (0.043)	0.064 (0.035)
	4	0.085 (0.040)	0.102 (0.054)	0.054 (0.030)	0.079 (0.054)	0.085 (0.044)
4	1	0.080 (0.042)	0.080 (0.039)	0.096 (0.035)	0.067 (0.031)	0.106 (0.052)
	2	0.071 (0.031)	0.076(0.051)	0.076 (0.034)	0.041 (0.024)	0.076 (0.039)
	3	0.090 (0.043)	0.078 (0.040)	0.090 (0.057)	0.044 (0.026)	0.084 (0.059)
5	1	0.084 (0.033)	0.090 (0.046)	0.085 (0.047)	0.087 (0.048)	0.084 (0.055)
	2	0.089 (0.052)	0.077 (0.043)	0.079 (0.049)	0.078 (0.033)	0.065 (0.044)
	3	0.091 (0.045)	0.109 (0.060)	0.078 (0.038)	0.082 (0.054)	0.078 (0.046)
	4	0.073 (0.037)	0.094 (0.054)	0.069 (0.030)	0.074 (0.046)	0.070 (0.044)
method : LWPR		SKI MEAN ERRORS (STANDARD DEVIATIONS)				
